# Comprehensive Self-Management of Heart Failure

**DOI:** 10.3390/jcdd12030107

**Published:** 2025-03-20

**Authors:** Shunsuke Kiuchi, Shigeto Tsukamoto, Rie Sato, Keisuke Hosono, Jun Okuda, Makoto Natsumeda, Mitsuharu Kawamura, Hideaki Tachibana, Takashi Okada, Takuro Takagi, Yasushi Taniguchi, Jiro Ando, Yutaka Koyama, Toshiro Shinke, Takanori Ikeda

**Affiliations:** 1 Department of Cardiovascular Medicine, Toho University Faculty of Medicine, 6-11-1 Omori-nishi, Ota-ku, Tokyo 143-8541, Japan; 2 Department of Cardiovascular Medicine, Toho University Omori Medical Center, Tokyo 143-8541, Japan; 3 Department of Medicine, Division of Cardiology, Showa University School of Medicine, Tokyo 142-8555, Japan; 4 Department of Cardiovascular Medicine, Tokyo Metropolitan Ebara Hospital, Tokyo 145-0065, Japan; 5 Department of Cardiovascular Medicine, Japanese Red Cross Omori Hospital, Tokyo 143-8527, Japan; 6 Department of Cardiovascular Medicine, Ikegami General Hospital, Tokyo 146-8531, Japan; 7 Department of Cardiovascular Medicine, Makita General Hospital, Tokyo 144-8501, Japan; 8 Department of Cardiovascular Medicine, Ota Ikegami Hospital, Tokyo 146-0082, Japan; 9 Department of Cardiology, Tokyo Heart Center, Tokyo,141-0001, Japan; 10 Department of Cardiology, Tokyo Shinagawa Hospital, Tokyo 140-8522, Japan; 11 Department of Cardiovascular Medicine, Jounanhukusiiryoukyokai Ota Hospital, Tokyo 143-0015, Japan; 12 Department of Cardiovascular Medicine, NTT Medical Center Tokyo, Tokyo 141-8625, Japan; 13 Department of Cardiovascular Medicine, Tokyo Kamata Hospital, Tokyo 144-0051, Japan

**Keywords:** heart failure, self-management, community heart failure management, prognosis

## Abstract

In response to the heart failure (HF) pandemic, it is important to introduce appropriate pharmacological and non-pharmacological treatments for HF patients. In addition, self-management of HF, including the continuation of appropriate pharmacological treatment, is also important. There have been many reports on self-management of HF. However, the effectiveness of patient education of HF is not consistent. One of the reasons may be whether the tools used are common or not. Moreover, unified systems of HF patient education and disease management in metropolitan areas are rare. We began joint HF patient education and disease management in the Tokyo southern medical district (JONAN Heart Failure Medical Collaboration: JHFeC). Patients undergo a multidisciplinary HF education program at JHFeC member hospitals to ensure that they are able to adequately record information on the self-management sheet. After discharge, the continuity of self-management will be evaluated, and further education will be provided if necessary. HF patient education is important even in severe HF requiring a left ventricular assist device, and such patient education needs to be provided appropriately in all manner of HF patients with stage A to D.

## 1. Introduction

The number of patients with heart failure (HF) is projected to reach approximately 1.3 million in Japan. This increase in the number of HF patients has been likened to an infectious disease and is referred to as an HF pandemic [1]. HF treatment has progressed dramatically in recent years with the advent of transcatheter minimally invasive treatments for structural heart disease and new medication treatments. These treatments shortened the length of hospital stays and reduced in-hospital mortality [2]. However, readmission rates of HF and mortality rates of 30 days or 1 year after discharge have not improved. Partly due to this result, the number of hospitalized HF patients is expected to continue to increase after 2030, with a peak expected in 2040 [3]. The most common cause of readmissions of HF is inadequate management of salt restriction, and when factors such as inadequate medication management, overwork, and physical and mental stress are added, more than half of the cases are caused by patient factors [4]. Therefore, appropriate self-management, including blood pressure (BP) management, may reduce readmission of HF. It is known that an increase in the number of readmissions of HF increases in-hospital mortality [5] and also increases post-discharge mortality [6]. In addition to advances in treatment, thorough and appropriate self-management is expected to prevent readmissions of HF and improve prognosis.

## 2. Self-Management System of HF

An appropriate patient educational program is important to promote self-management of HF. The HF educational program needs to be provided by multiple professions, and is time-consuming. Because of this, an HF education program was provided at each medical institution. Moreover, these interventions need to be ongoing. The improved self-management of HF resulting from these HF educational programs has also been recommended by the European Society of Cardiology [7]. Previous studies have shown that continuous interventions for patients or their caregivers can improve HF self-management ability [8]. However, intervention by nurses specializing in management of patients with HF or experts did not improve HF prognosis [9,10]. Other previous studies revealed no significant association between HF self-management and HF exacerbation [11]. On the other hand, it has also been reported that appropriate patient education can lead to improved prognosis [12,13]. The effectiveness of patient education of HF is not consistent (Table 1), and one of the reasons may be whether the tools used are common or not. To improve these, it is necessary to provide common education and programs aimed at improving HF self-management ability at multiple institutions.

Another obstacle to self-management is that many patients with HF need caregivers to help them manage their disease. Caregivers can be partners, family members, friends, or neighbors, and the patients rely on them for unpaid support. With advances in medications and invasive, non-invasive treatments for HF, the responsibilities of caregivers are expanding. Thus, caregivers need more education about HF, and their willingness to cooperate in HF care is important. However, it has been reported that caregiver contribution to self-care of HF is insufficient [14]. Approximately one-third of staff at medical institutions with nursing homes were not yet prepared to cooperate [15]. One of these factors is the caregiver knowledge and understanding of HF. Even education by watching a 6-min YouTube video explaining HF and community cooperation improved cooperation [15]. In addition, the health-related quality of life in the caregiver also affects cooperation, especially in the case of caregivers in the home [16]. Age and mental state (depression) of the caregiver should also be taken into consideration [17]. In order to properly manage HF self-management, it is important to consider these factors and receive support from appropriate caregivers.

## 3. HF Medical Cooperation in the Tokyo Southern Medical District

Patients can learn a lot of information from HF education programs offered by medical institutions, as well as from care managers and other professionals who gather information from various sources, including social media. However, if patients understand the same information differently depending on how it is presented, patient education will not be useful. Therefore, we have initiated unified patient education at local medical institutions (JONAN HF Medical Collaboration: JHFeC). The Tokyo southern medical district has two medical-school-affiliated hospitals and many core hospitals that treat HF, and a meeting to start unified patient education among these hospitals was held in December 2021. With the support of many core hospitals with more than 200 beds, JHFeC concept began in April 2022 at 10 hospitals (Toho University Omori Medicine Center, Showa University Hospital, Tokyo Metropolitan Ebara Hospital, Tokyo Kamata Medical Center, Japanese Red Cross Omori Hospital, Ikegami General Hospital, Makita General Hospital, Ota Ikegami Hospital, NTT Medical Center Tokyo, and Tokyo Kamata Hospital). The Tokyo southern medical district includes Ota-ku and Shinagawa-ku, and five medical associations are active (Ota-ku: Kamata Medical Association, Omori Medical Association, Denenchofu Medical Association; Shinagawa-ku: Shinagawa Medical Association, Ebara Medical Association). In collaboration with these medical associations, JHFeC activities began after a kick-off meeting in September 2022. Currently, JHFeC is active in a total of 13 hospitals, including 3 more hospitals (Tokyo Heart Center, Tokyo Shinagawa Hospital, and Jounanhukusiiryoukyokai Ota Hospital). In medical collaboration with multiple professions, collaboration is conducted within each hospital with many professions (certified HF educators, nurses, pharmacists, rehabilitation instructors, nutritionists, etc.). Additionally, in the paramedical area, collaboration is important not only within each hospital but also between hospitals, and in June 2023, JHFeC medical staff collaboration began. It has also been reported that patient education, including cardiac rehabilitation, is highly cost-effective in terms of medical expenses [18]. It is expected that these activities will prevent re-hospitalization for HF and improve prognosis, resulting in high cost efficiency in terms of medical expenses.

## 4. Characteristics of JHFeC in Urban Medical Collaboration

HF medical collaboration is being carried out in many regions [19]; however, it is difficult to operate such medical collaboration in metropolitan areas. It has been reported that hospital cardiologists and general practitioners (cardiology and non-cardiology) have different perspectives on patient management [20]. Another factor may be that in large metropolitan areas, there are many core hospitals in the same area, and each core hospital has its own patient education and management programs. There are also issues such as differences in emergency response methods and the time it takes to collect past patient information when a patient is hospitalized at a hospital other than the one where they normally receive medical care. As a result, in the case of HF medical collaboration in metropolitan areas in Japan, Osaka Stops HEart Failure (OSHEF), which operates Heart Note^®^, is well-known; however, in Tokyo, there are only small-scale collaborations. The Tokyo southern medical district where JHFeC is active has a population of over 1.1 million, and the population is expected to continue to increase [21]. Another characteristic of this medical area is that the regional medical completion rate from the acute to chronic phase is high, at approximately 70–80%. As mentioned above, in HF medical collaboration, each medical institution needs to provide the same patient education. In addition, the same evaluation methods need to be used to understand patient conditions, and the same management needs to be based on those evaluations. Medical areas with a high rate of the regional medical completion may find it easier to unify their standards. JHFeC have started unified patient education and disease management using Heart Note^®^ (Medical Healthcare X-Innovation LAB, Osaka, Japan).

## 5. JHFeC Activities and Heart Failure Self-Care Management

**Figure 1:**
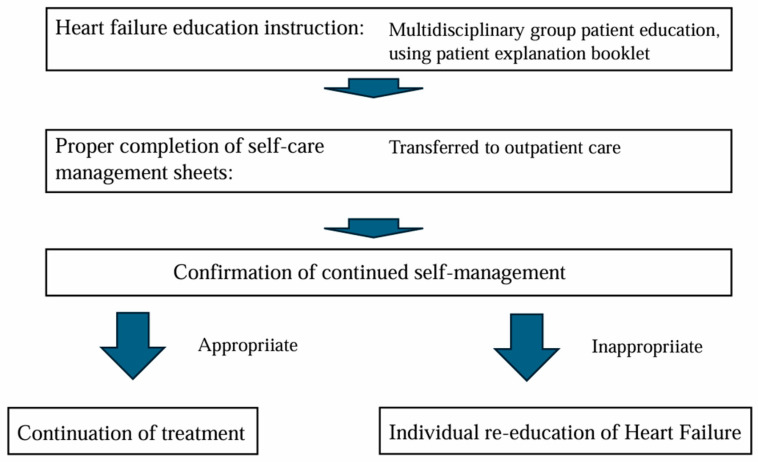
A flowchart of the HF educational program. Sufficient time is spent on HF patient education during hospitalization by multiple professions, and after many checks to ensure that patients are able to fill out their self-management sheet adequately, patients are transferred to outpatient care. After discharge, the continuity of self-management will be evaluated, and further education will be provided if necessary.

**Figure 2:**
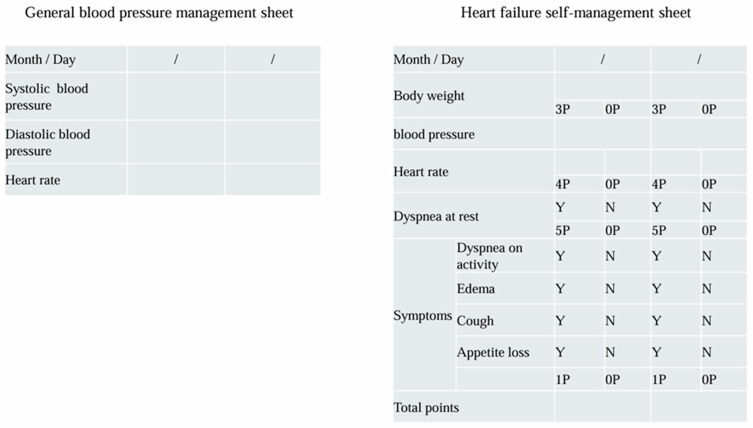
Comparison of general blood pressure management sheet and heart failure self-management sheet. The number of factors in heart failure self-management sheet to be recorded does not increase significantly compared to a blood pressure diary. Y: Yes, N: No.

Heart Note^®^ is comprised of a patient explanation booklet, self-management sheet, and a medical collaboration form. The HF education program needs to be provided by multiple professions, and it takes a lot of time, including checking the accurate entries in the self-management sheet. It is not easy to provide this education program during outpatient care and to check the entries in the self-management sheet. Therefore, many JHFeC member hospitals provide multidisciplinary HF education programs to hospitalized HF patients using the patient explanation booklet. In our hospital (Toho University Omori Medical Center), sufficient time is spent on HF patient education during hospitalization, and after many checks to ensure that patients are able to fill out their self-management sheet adequately, patients are transferred to outpatient care. HF patient education is conducted in small group classrooms; however, there are differences in the level of patient understanding of HF and their own understanding of the current state of HF. For this reason, we also check each patient’s level of understanding individually when checking to make sure they have filled out their self-management sheet appropriately. Moreover, after discharge, the continuity of self-management will be evaluated, and further individual education will be provided if necessary. A flowchart of the HF educational program is shown in Figure 1. In managing hypertension, one of the major causes of HF, it is important to keep a BP diary. In BP diaries, systolic and diastolic BP, as well as pulse rates in the morning and evening, are often recorded. The self-management sheet attached to the Heart Note^®^ used in JHFeC records BP, pulse rate, weight, and the presence or absence of HF symptoms [12] (Figure 2).

The number of factors to be recorded does not increase significantly compared to a BP diary; therefore, if patients are able to record in a BP diary, it is entirely possible to record in a self-management sheet. It has also been reported that the use of this self-management sheet reduces re-hospitalization for HF by approximately 50% [12]; therefore, it is important to first take into account the habit of self-management, including strict BP control by recording BP values. JHFeC provides patient education and disease management using unified materials, and regional collaboration is established among multiple medical institutions in metropolitan areas. Past medical behavior affected their decision to influence care-seeking delay or avoidance in patient HF management [22], and the other purpose of JHFeC activities is to link appropriate self-management to appropriate medical consultation. The Kansas City Cardiomyopathy Questionnaire (KCCQ) score is also widely used as an existing scale for assessing HF symptoms. It is necessary to use such indices in the future to evaluate the usefulness of JHFeC activities [23].

An HF education program to help patients learn HF self-management is mainly provided by “Certified Heart Failure Educator (CHFE)” in each institution. When providing medical care through an HF team involving multidisciplinary collaboration, it is necessary for all members to acquire common knowledge about HF. As a measure to achieve this, the Japanese Circulation Society took the lead in creating the CHFE qualification for the multidisciplinary personnel responsible for team medical care. Medical staff who have a certain level of knowledge related to HF and medical education, as determined by report and written examinations, are able to work as a CHFE. We would also like to emphasize that JHFeC is able to provide high-quality HF education through activities centered around CHFE. Another characteristic of JHFeC is that opinions are exchanged not only between doctors at each institution, but also between CHFEs. This exchange of opinions takes place monthly using online systems. Education and intellectual level are also considered to be related to HF self-management [24]. In addition, patient background, such as age and mental conditions, is also an important factor [25]. HF is known to be prone to induce depression, and it has been revealed that spouses of HF patients are also prone to develop depression [26]. Depression strongly affects the continuation of self-management [27]. The impact on self-management may be one of the factors that worsen the prognosis of HF [28]. CHFE includes not only nurses but also many other professions; thus, it can provide continuous support for these problems.

## 6. Issues in Regional Collaboration for HF

Throughout the HF pandemic, seamless HF treatment through a regional comprehensive system including multi-disciplinary collaboration is attracting attention, and many societies related to cardiovascular medicine are holding symposiums. Two points that often come up in these discussions are: (1) whether to use paper or digital media such as a mobile phone application, and (2) whether such media should be standardized nationwide or unique to the region. Paper-based assessments can be completed at any time with writing utensils; however, they need to be carried around. On the other hand, digital media have the advantage of being easy to carry and easy to display past data in chronological order; however, if you are not familiar with digital media, it may not be easy to operate them. Self-management is particularly difficult for elderly HF patients [29], and it is essential that the information is adequately recorded and used, whether on paper or digital media. It has also been reported that continuous intervention by telephone improves self-management [30]), and it may be important to designate their caregivers who will accurately manage the patient together with the patient, regardless of the management media. Furthermore, the etiology of HF differs from country to country [31], and this is true even within Japan [22]. In this regard, management media that take advantage of the characteristics of each region may be useful. On the other hand, it has been pointed out that hospital cardiologists and general practitioners have different goals, such as the medical information they require [20]. If the management medium differs from region to region, the management objectives will also differ if a patient moves to a different region, for example by relocating. If management goals differ, doubts will arise about the patient HF self-management, leading to a deterioration in the management situation. Therefore, it is essential to make full use of the management media currently in use in the area and continue appropriate management.

## 7. Self-Management in Severe Heart Failure

The importance of self-management in HF is not only about its effectiveness in preventing re-hospitalization due to HF. Self-management is also extremely important when managing a left ventricular assist device (LVAD), and support from caregivers is equally important. In Japan, heart transplants began under legal provisions in 1997, and the number of donated organs has been gradually increasing, following the 2010 revision of the Organ Transplant Law. However, the number of donated organs is not enough to meet demand, and the waiting period for a heart transplant exceeded 1500 days in 2019. It is also necessary to pay attention to maintaining hemodynamics during this waiting period, and the use of implantable LVAD as a bridge to transplantation (BTT) has begun. However, because implantable LVAD cannot be used for patients who are not eligible for heart transplants, they are often forced to abandon this life-saving treatment. Even now, the waiting times for heart transplants remain long. For these reasons, in Japan, in addition to BTT, the use of LVAD as Destination Therapy (DT-LVAD) for long-term home support without the need for a heart transplant became eligible for insurance coverage from May 2021 [32]. The Japanese registry for mechanically assisted circulatory support (J-MACS) reported that the number of LVAD placements in Japan is also increasing [33]. The self-management of LVAD requires sufficient self-management ability, as defined by a mini-mental state examination score of 24 or more and a trail-making test B score of 300 s or more. In addition, patients will need to live with their caregivers for six months or more; thus, adequate education and understanding of both patients and caregivers is essential. Adverse events such as driveline infections are a concern with LVAD [34]; however, the risk can be reduced with proper management. In this respect, it is also important for CHFE to provide appropriate education to patients and caregivers.

## 8. Conclusions

In HF regional collaboration, joint activities for patient education and disease management are important. JHFeC has begun joint activities in the Tokyo southern medical district. HF patient education and disease management are conducted in each region; however, JHFeC will provide useful information for these activities through regional collaboration in metropolitan areas.

## Figures and Tables

**Table 1:** Characteristics of Studies Regarding self-management of HF.

Reference No.	Subjects	No. of Patients (% of Male)	Mean Age (Years)	Method	Duration	Main Results
[8]	HF patients with insufficient self-care and their caregivers.	510 (58%)	74	Arm 1 (MI only for patients) Arm 2 (MI for patients and caregivers) Arm 3 (usual care).	12 months	Self-care management in Arms 1 and 2 were significantly higher than in Arm 3
[9]	mild HF (50%) and moderate to severe HF (50%)	1023 (62%)	71	Control group (usual care) and 2 support groups (additional basic or intensive support by a nurse)	18 months	Disease management by a nurse did not reduce death and hospitalization of HF
[10]	HF reduced Ejection Fraction	5647 (67%)	63	Quality improvement intervention or usual care	12 months	There was no significant difference in time to first HF rehospitalization or death between 2 groups
[11]	Chronic HF	97 (54%)	81.4	Usual care and evaluated using the Self-Care of Heart Failure Index	6 months	There was no significant association between the Self-Care of HF Index and HF exacerbation
[12]	HF	306 (55.9%) After propensity-score matching	78	Comparison of before and after induction of the self-management system	12 months	The induction of the self-care system improved the prognosis of HF
[13]	HF	598 (57%)	79	Control: before program implementation Phase 1: transitional HF management program implementation Phase 2: following program revision and regional dissemination	12 months	HF readmission rates gradually decreased over Phases 1 and 2 (*p* < 0.05)
